# IgG4‐Related Disease: Emerging Roles of Novel Genetic Variants, Immune Cell Subsets and Therapeutic Targets

**DOI:** 10.1111/all.16686

**Published:** 2025-08-11

**Authors:** Louisa Tedesco, Syed B. Ali, Baran Erman, Safa Baris, Harshita Pant, Pravin Hissaria, Yelda Bilginer, Damon J. Tumes, Gökhan Cildir

**Affiliations:** ^1^ Centre for Cancer Biology University of South Australia and SA Pathology Adelaide Australia; ^2^ Department of Clinical Immunology and Allergy Flinders Medical Centre Bedford Park South Australia Australia; ^3^ School of Medicine and Biomedical Sciences Flinders University Bedford Park South Australia Australia; ^4^ Can Sucak Research Laboratory for Translational Immunology Hacettepe University Ankara Türkiye; ^5^ Institute of Child Health Hacettepe University Ankara Türkiye; ^6^ Faculty of Medicine, Division of Pediatric Allergy and Immunology Marmara University Istanbul Türkiye; ^7^ Isil Berat Barlan Center for Translational Medicine Istanbul Jeffrey Modell Foundation Diagnostic Center for Primary Immune Deficiencies Istanbul Türkiye; ^8^ Discipline of Medicine, Adelaide Medical School, Faculty of Health and Medical Sciences University of Adelaide Adelaide South Australia Australia; ^9^ Adelaide Centre for Epigenetics, School of Biomedicine University of Adelaide Adelaide South Australia Australia; ^10^ Adelaide Medical School, University of Adelaide, Adelaide, Australia; Division of Human Immunology, SA Pathology, Adelaide, Australia; Clinical Immunology and Allergy Royal Adelaide Hospital Adelaide Australia; ^11^ Department of Pediatric Rheumatology, Faculty of Medicine Hacettepe University Ankara Türkiye

**Keywords:** fibrosis, IgG4, IgG4‐related disease, immune dysregulation, inebilizumab

## Abstract

IgG4, the least abundant IgG subclass in humans, is increasingly recognised for its involvement in allergic and autoimmune pathologies. Its unique properties, such as the tendency to form half‐molecules (one heavy chain and one light chain) and its generally non‐inflammatory nature, distinguish it from other IgG subclasses. Its role in immune dysregulation is further underscored by a distinct disease entity called IgG4‐related disease (IgG4‐RD), a systemic fibroinflammatory disorder characterised by IgG4^+^ plasma cell tissue infiltration, storiform fibrosis and obliterative phlebitis, reflecting a dysregulated immune response affecting multiple organs. This review examines the current understanding of IgG4, its emerging roles in immune dysregulation and IgG4‐RD, including clinical manifestations and treatment options. We also discuss the recent advances in understanding the genetic underpinnings of IgG4‐RD, highlighting the significance of germline gene variants and implicated immune cell types. Finally, we explore future research directions, emphasising the need for deeper insights into pathogenesis, specific biomarkers and optimised treatment strategies.

## 1 Introduction

IgG4 is the least abundant IgG in human serum (accounts for 4%–5% of total IgG [1]) and up to 10% circulates as ‘half‐molecules’. Although IgG4 is not universally present across all species [2], a large body of evidence supports that it has a significant role in disease conditions with allergic inflammation [3] and autoimmunity [4] among many others. IgG4 possesses a unique feature known as ‘Fab‐arm’ exchange. When IgG4 molecules undergo Fab‐arm exchange, heavy‐light chain pairs (half‐molecules) can dissociate; one half‐molecule can then associate with another half‐molecule from a different IgG4 molecule to form a complete IgG4 dimer. Gaining bi‐specificity (two different antigen‐binding sites) reduces the antigen cross‐linking capacity of IgG4 and inhibits the activation of IgE responses [5], which are the main drivers of mast cell [6] and basophil‐mediated allergic inflammation. IgG4 has also been reported to have low binding capacity to C1q and Fcγ receptors [2]. Altogether, these properties of IgG4 partly explain why IgG4 antibodies are generally regarded as non‐inflammatory. Class‐switching to IgG4 is mediated by several stimuli, including prolonged antigenic stimulation, IL‐4 and IL‐10. Notably, an increase in allergen‐specific IgG4 is well correlated with immune tolerance and decreased hypersensitivities [7, 8].

### 1.1 IgG4‐Related Disease (IgG4‐RD)

IgG4‐Related Disease (IgG4‐RD) is a rare and severe systemic fibroinflammatory disorder that predominantly affects older adults, with a peak incidence in the fifth to seventh decades of life, and exhibits a significant male predisposition, with men developing the disease more than twice as often as women [9]. Histopathological features include dense lymphoplasmacytic infiltration of IgG4^+^ plasma cells, obliterative phlebitis (inflammation and blockage of veins by a dense buildup of immune cells) and storiform (weave‐pattern) fibrosis [10]. Currently, there are four clinical phenotypes identified based on shared observations between affected organs: pancreato‐biliary lesions (Group 1), retroperitoneal fibrosis/aortitis lesions (Group 2), head and neck lesions (Group 3) and Mikulicz disease that is accompanied by systemic lesions (Group 4) [11].

There is also an increased recognition of IgG4‐RD in the younger patient cohort. Notably, paediatric IgG4‐RD exhibits characteristics that differ from the adult form, particularly in terms of sex distribution and involved organs. Unlike adult cases, paediatric IgG4‐RD lacks a clear male predominance, with surface organ involvement, such as ophthalmic diseases, being more frequently affected [12].

IgG4‐RD is generally a chronic condition with a slow and indolent progression. Symptom duration varies widely depending on the affected organs and the stage of the disease at diagnosis [13]. Symptoms can be intermittent or persist for months to years before diagnosis, and patients often present with organ enlargement or tumour‐like masses [14]. As some patients may remain asymptomatic for extended periods, there may be a delay in diagnosis, and lesions may lead to progressive organ damage. Additionally, due to tumefactive lesions in affected organs, IgG4‐RD can resemble other infiltrative diseases and malignancies [15]. Hence, the difficulty of diagnosis and the potential for misdiagnosis of IgG4‐RD are serious concerns. Furthermore, while an elevated serum IgG4 is associated with IgG4‐RD, IgG4 levels can vary among the different extents of organ involvements and thus cannot serve as a reliable and specific single diagnostic marker [16]. Glucocorticoids are the mainstay treatment for IgG4‐RD and are the cornerstone for inducing remission. Tapering regimens are successful, with glucocorticoid‐based first‐line regimens showing a 97% response rate [17]. However, the urgent need to develop an approved therapy for IgG4‐RD remains, as glucocorticoids are not FDA‐approved specifically for the disease. Relapse rates and corticosteroid toxicity are also causes for concern, particularly as patients are often older with underlying comorbidities at diagnosis. Recently developed B cell depletion therapies, such as inebilizumab [18], have shown marked reduction of serum IgG4 and disease flare risk, as well as overall clinical improvement, and have been recently approved by the FDA. For disease maintenance, combination with conventional synthetic disease‐modifying antirheumatic drugs (csDMARDS), such as azathioprine, methotrexate and mycophenolate mofetil, is often used for maintaining remission and reducing chronic corticosteroid burden and associated toxicity [19]. IgG4‐RD places a substantial burden not only on patients and their families but also on the healthcare sector at large. Healthcare costs are substantially higher for IgG4‐RD patients due to frequent comorbidities such as hypertension, diabetes, infections and steroid‐related toxicities [20]. Additionally, due to concerns of infiltrative diseases, multiple biopsies and imaging may be required, further amplifying healthcare costs and leading to high emotional stress for the patients. Recurrence rates of IgG4‐RD post‐remission therapy are approximately 40% [21], increasing hospital readmission rates and rehabilitation costs. Patients experience fear of worsening symptom flare‐ups and distress due to the general lack of awareness within the medical community.

### 1.2 Diagnostic Criteria

There are three diagnostic criteria for IgG4‐RD: Umehara [22, 23], Okazaki Criteria [24] and the American College of Rheumatology/European League Against Rheumatism [11]. In a recent comparative analysis, the Umehara demonstrated the highest sensitivity, while all three criteria showed consistent specificity [25]. In 2012, Umehara et al. were the first group to establish a comprehensive diagnostic criterion for IgG4‐RD almost a decade after its description as a distinct disease entity [23]. This criterion provided a ‘definite’ diagnosis of IgG4‐RD if two of three major items were fulfilled: internal organ involvement as manifested by swelling, serum IgG4 levels > 135 mg/dL and histological findings of plasmacyte infiltration with > 10 IgG4^+^ cells per high‐powered field, and a > 40% ratio of IgG4^+^/IgG cells with accompanying fibrosis [23]. A 2020 Revised comprehensive diagnostic (RCD) criteria was published thereafter [22]. The RCD criteria similarly had three items; however, item 1, a singular lymph node swelling, has been incorporated as exclusion, while item 3 has histopathology further subdivided, for which two of three were required: dense lymphocytic and plasma cell infiltration, plasmacyte infiltration with > 10 IgG4^+^ cells per high‐powered field and a > 40% ratio of IgG4^+^/IgG cells, and fibrosis, especially storiform fibrosis or obliterative phlebitis [22]. Given the heterogeneity in IgG4^+^ and organ involvement, the RCD criteria incorporate this in accordance with seven IgG4‐related organs: pancreatic, lacrimal and salivary gland, renal, biliary system, ophthalmic, respiratory and retroperitoneal and/or periarterial. The RCD also subgroups the diagnoses as definite (all three items), probable (Item 1 and 3) and possible (Item 1 and 2). Okazaki, similarly, has three items and places an emphasis on histology, including diagnosis possible on all four features: lymphocytic and plasmocytic infiltrate with fibrosis, without neutrophil infiltrate, and the remaining three features like the Umehara histology item 3 criteria. The 2019 ACR/EULAR has a comprehensive point system after a list of exclusion criteria (clinical, serologic, radiological, histological and specific disease entities) with histopathology, immunostaining, as well as key clinical and radiological features: head and neck (lacrimal, parotid, sublingual and submaxillary salivary glands), chest, pancreas and biliary duct, kidney and retroperitoneum [11]. A score at or greater than 20 is most likely for an IgG4‐RD diagnosis [11].

### 1.3 Epidemiology

IgG4‐RD was first recognised as a unique clinicopathological entity termed ‘systemic IgG4‐related autoimmune disease’ in 2003 (Table 1). Japanese researchers characterised pancreatic lesions in autoimmune pancreatitis (AIP) patients that were unrelated to AIP pathology. Rather, these lesions were associated with an IgG4‐related systemic autoimmune disease with extensive organ involvement [28]. IgG4‐RD can be more broadly described as the aberrant infiltration of IgG4‐restricted plasma cells into a variety of organs, most commonly the pancreas and lymph nodes [39]. Its prevalence seems to be low, unknown or underestimated [40] because rigorous epidemiological studies of IgG4‐RD are not available [39]. The Japanese Ministry of Health, Labour and Welfare [41] reports 330–1300 newly diagnosed cases per year, with a frequency of 6.3 cases per 100,000 individuals [42].

**TABLE 1:** Timeline of IgG4‐RD major discoveries and other milestones.

1961	Pancreatitis with chronic sclerosis and hypergammaglobulinemia first described [26]
1995	Autoimmune pancreatitis (AIP) put forward as a clinical entity [26]
2001	Discovery of IgG4^+^ plasma cells in pancreatic lesions and elevated serum IgG4 in AIP [27]
2003	Systemic IgG4‐related autoimmune disease described as a distinct clinicopathological entity [28]
2006	Mikulicz's disease is conceptualised as systemic IgG4 plasmocytic syndrome [29]
2009	IgG4‐RD linked with a newly recognised subset of non‐infectious aortitis [30]
2010	Riedel's thyroiditis and multifocal fibrosclerosis added to IgG4‐related systemic disease spectrum [31]
2010	B‐cell targeting to halt IgG4 production and reduce inflammation first hypothesised [32]
2011	International symposium establishes ‘IgG4‐related disease’ as the accepted nomenclature [33]
2011	Comprehensive diagnostic criteria for IgG4‐RD published [23]
2012	IgG4‐RD Responder Index developed [34]
2015	Rituximab proposed as an effective treatment as well as a glucocorticoid‐sparing agent [35]
2019	ACR/EULAR consensus statement and classification criteria published [36]
2020	Revised comprehensive diagnostic criteria for IgG4‐RD published [22]
2023	Novel Patient‐Reported Outcome Measure in IgG4‐RD published [37]
2024	Successful Phase III trial of Inebilizumab for IgG4‐RD treatment [18]
2024	Complement activation declared ‘likely’ to be involved in IgG4‐RD pathophysiology [38]
2025	Inebilizumab approved by the FDA as the first and only therapy against IgG4‐RD

**FIGURE 1:**
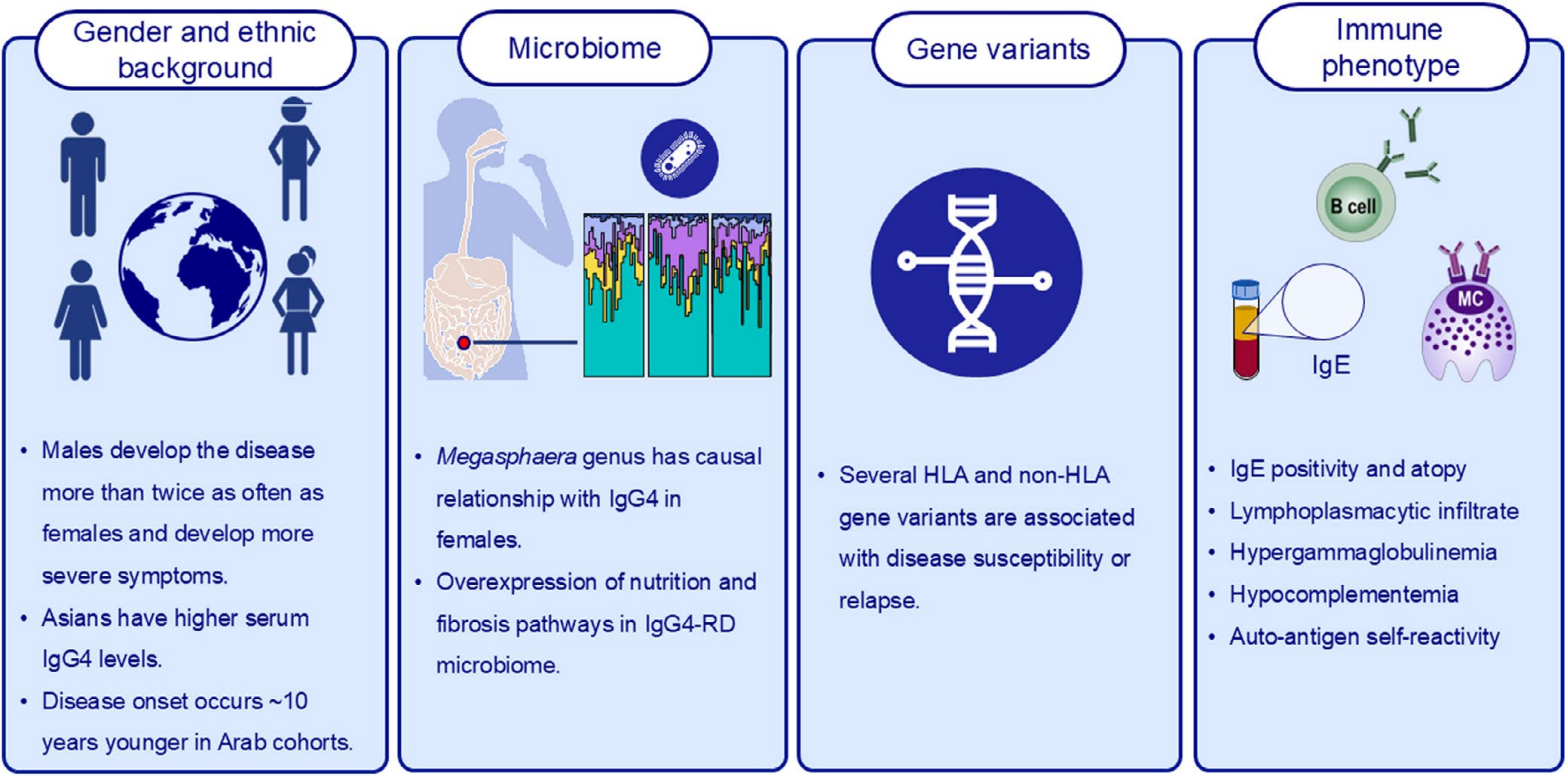
There are four main factors influencing IgG4‐RD heterogeneity: gender and ethnic background, the microbiome, gene variants and immune phenotype.

Disease heterogeneity may be attributed to genetic or non‐genetic factors (Figure 1). For example, diagnosis occurs around 60 years of age globally, apart from Arab cohorts, where the age of onset is approximately 10 years younger [43]. Males are more frequently affected and tend to have a worse prognosis, except in cases of head and neck involvement [42]. For females, disease‐onset generally occurs at a younger age and allergic disease is more prevalent. Lower peripheral eosinophils, C‐reactive protein (CRP) and serum IgG4 levels are associated with a better prognosis in females [44]. Regional variations in disease expression have also been reported. A recent retrospective follow‐up study highlights the absence of pancreatic involvement and increased renal and retroperitoneal involvement in Arab populations [43], whereas salivary gland and pancreatic involvement are more frequently observed across cohorts from the United States (US) and Japan [45, 46].

### 1.4 IgG4 Antibody Pathogenicity and Hypocomplementemia

The aetiology and pathogenesis of IgG4‐RD are poorly understood, largely because it is difficult to determine whether IgG4 is driving the disease or regulating a separate autoimmune or allergic process mainly driven by other immune cell populations. IgG4 (especially in its bispecific, monovalent composition [47]) is regarded as an anti‐inflammatory antibody, partly because of its limited affinity for human Fc receptors [48]. In vitro studies with IgG1, the murine homologue of IgG4, suggest that IgG4 can block the hexamerisation of other IgG‐class antibodies required for C1q binding and subsequent complement activation through steric hindrance [49]. One study also reported that IgG4 binds well to FcγRIIB, an inhibitory receptor, which supports the role of IgG4 in blocking the complement response [50]. On the contrary, another study suggests that the involvement of IgG4‐mediated complement activation in disease pathology is possible. Using glycol‐engineered monoclonal preparations of IgG4, Oskam et al. demonstrated that IgG4 can activate complement at high antigen and high antibody concentrations via the classical pathway [47]. Katz et al. also suggest that IgG1 and IgG4 can form mixed immune complexes to activate the classical pathway [38]. Their single‐centre cross‐sectional study of 279 patients from the Mass General Hospital IgG4‐RD cohort, which is among the largest in the world, assessed disease characteristics of patients with hypocomplementaemia. Hypocomplementaemia occurs when the complement pathway is hyperactivated and complement proteins deposit on the surface of target cells, reducing their concentration in the blood. In an IgG4‐RD context, hypocomplementaemia was traditionally associated with IgG4‐related tubulointerstitial nephritis, the most common form of IgG4‐related kidney disease [51]. Hypocomplementaemia is also observed in other autoimmune and rheumatic diseases, such as systemic lupus erythematosus (SLE) [52]. Katz et al. identified a possible link between complement activation and disease severity, with low serum levels of complement components C3 and C4 in 32% of their IgG4‐RD patient cohort [38]. Hypocomplementaemic patients were found to have higher disease activity scores, number of organs involved, serum IgG4 levels and relapse rates than those without hypocomplementaemia [38].

### 1.5 Autoantibodies

One of the ways in which the central role of B cells in IgG4‐RD has been demonstrated is through the presence of autoantibodies in the disease. Within the context of IgG4‐related autoimmune pancreatitis, antibodies against lactoferrin, carbonic anhydrase, pancreas secretory trypsin inhibitor, amylase‐alpha, heat shock protein and plasminogen‐binding protein have been identified [4]. However, autoantibody sensitivity and specificity are not consistent across autoantigens and other organ manifestations [53]. Liu et al. identified autoantibody responses against laminin 511‐E8, prohibitin, annexin A11 and galectin‐3 within a clinically diverse IgG4‐RD cohort from the US [54]. The study indicated that having one or more types of autoantibodies was linked to more severe disease, including higher IgG subclass levels, hypocomplementemia and a greater chance of visceral organ involvement [54].

### 1.6 Microbiome Signature

Koshida et al. sought to examine the causal relationship between the gut microbiome and serum IgG4 levels in the general population [55]. Their study highlighted the variation of the gut microbiome between sexes and hypothesised that gender‐related differences in IgG4‐RD onset may reflect the sex‐delineated composition of the gut microbiome and its influence on serum IgG4 levels. Causal inference in women showed that *Megasphaera* increased IgG4 levels; however, no bacterial genera presented a causal relationship with serum IgG4 levels in men [55]. *Megasphaera* composition is a significant characteristic that differentiates males and females, and the genus comprises anaerobes which can metabolise short‐chain fatty acids (SCFAs) [55]. While the study suggested that SCFAs may influence IgG4 production through their impact on inflammatory cytokines, they concluded that a causal relationship between identified genera and IgG4 could not be definitively established [55]. Plichta et al. established that the microbiomes of systemic sclerosis and IgG4‐RD were distinctly separate from healthy individuals [56]. Pathways related to nutrition (ethanolamine metabolism) and fibrosis (hydroxyproline metabolism and fibronectin binding) were found to be overexpressed in disease cohorts. This indicates that the microbiome may have enhanced capabilities for nutrient acquisition, tissue interaction and potential virulence in autoimmune patients [56]. Significantly overabundant *Streptococcus* species and numerous opportunistic pathogenic *Clostridium* were identified in their disease cohorts, whereas butyrate‐producing species were depleted compared to healthy controls [56]. Strain‐level analysis showed *Clostridium boltae* encoding a strain‐specific cysteine uptake and metabolism locus preferentially colonised autoimmune patients. This could contribute to the perpetuation of dysbiosis, altered immune responses and the progression of autoimmune diseases [56]. Overall, modulating the immune response through the gut microbiome presents a potential therapeutic approach for other autoimmune conditions, including IgG4‐RD.

### 1.7 Genetic Aetiology

Despite a large research focus on the underlying immunological mechanisms of IgG4‐RD, there is a paucity in the literature regarding the genetic aetiology of IgG4‐RD. Recently, risk alleles within the human leukocyte antigen (HLA) are being increasingly described. Terao et al. [57] conducted a genome‐wide association study (GWAS) of IgG4‐RD, which reported three susceptibility loci: *HLA‐DRB1*, *HLA‐A* and *FCGR2B*. The *HLA‐DRB1‐GB‐7‐Val* gene variant is particularly interesting, as it appears to significantly affect disease severity and treatment response in IgG4‐RD. Yamamoto et al. [58] observed a trend towards an increased frequency of patients carrying the *HLA‐DRB1‐GB‐7‐Val* allele requiring higher doses of corticosteroids and higher relapse rates. Tsuchiya and Kawasaki [59] highlight that the HLA‐DRB1*09:01‐DQB1*03:03 haplotype reported by Terao et al. was not only found to be decreased in patients with IgG4‐RD but may also be indicative of proclivity towards other types of autoimmune disease (AID). A Chinese retrospective cohort study showed that 14.8% of IgG4‐RD patients had AID in their family history, with younger age of disease onset and higher frequency of anti‐nuclear antibody positivity in the AID‐positive group [13]. Determining shared underlying mechanisms and risk alleles between well‐studied AIDs and IgG4‐RD would serve as an important catalyst to elucidate the genetic aetiology of IgG4‐RD.

In recent years, several non‐HLA gene variants have also been identified as risk alleles for IgG4‐RD (Table 2). Newman et al. applied WGS to'family screen' a 48‐year‐old male with recently diagnosed IgG4‐RD [73]. They identified a rare unreported heterozygous single base deletion in the *FGFBP2* gene, which causes a frameshift mutation in the protein‐coding sequence for FGFBP2. They suggest that an aberrant FGFBP2 secreted by CD4^+^ cytotoxic T lymphocytes (CTLs) may enhance their function. Liu et al. identified *IKZF1* ^R183H^ and *UBR4* ^C4179*^ variants in familial IgG4‐RD, shared by a father and both daughters [65]. *IKZF1* ^R183H^ is a gain‐of‐function mutant located in the DNA‐binding domain, which hyperactivates the *FYN* promoter in T cells. *UBR4* ^C4179*^ truncates the E3 ubiquitin‐protein ligase, likely causing a loss of function. Both effects were reported to synergistically lower the T activation threshold, which was supported by T cells from affected family members showing hyperresponsiveness to stimulation. *IKZF1* and *UBR4* gene variants support a close link between independent T cell activation and enhanced Th2 differentiation in disease pathogenesis. However, further studies are needed to confirm this association. Medhavy et al. [72] identified *TNIP1* ^*Q333P*^ as an ultrarare heterozygous variant in autoimmune patients with elevated IgG4. Multifocal lymphocytic sialadenitis (salivary gland inflammation) was observed in mice with the orthologous *Tnip1* ^*Q346P*^ variant, as well as the development of spontaneous germinal centres and expansion of age‐associated B cells, plasma cells and follicular and extrafollicular helper T cells. Fujibayashi et al. [60] high‐throughput sequenced 27 Japanese patients with autoimmune pancreatitis (AIP), which is classified as an IgG4‐RD Group 1 manifestation. Nine candidate genes were significantly associated with AIP susceptibility, among them *P2RX3* and *TOP1*. DNA topoisomerase is encoded by *TOP1* and is associated with the development of several autoimmune diseases [74]. Insulin release is controlled by positive autocrine signalling from the ligand‐gated ion channel P2X purinoceptor 3 (P2X3R) encoded by *P2RX3*. Notably, ATP released from damaged and inflamed tissues engages P2X receptors (including P2X3R) expressed on primary afferent neurons to drive nociceptive signalling and chronic pain [75], a symptom commonly experienced by IgG4‐RD patients with chronic pancreatitis. Table 2 provides a comprehensive summary on non‐*HLA* variants associated with IgG4‐RD, including clinical phenotypes.

**TABLE 2:** Non‐HLA gene variants associated with elevated IgG4 levels or IgG4‐RD.

Gene	Variant(s)	Phenotype
*CACNA1C* [60]	c.5996delC	Identified link with extra‐pancreatic lesions
*CTLA4* [61, 62]	+49A/A +6230A/A	Enhanced relapse risk in AIP, CTLA4 serum levels significantly higher in AIP
*CXCR3* [60]	c.630_631delGC	Identified link with extra‐pancreatic lesions. Gene has a role in plasmacytoid dendritic cell‐driven AIP [63]
*FCGR2B* [57]	rs1340976	Swollen organs and IgG4 concentration at diagnosis
*FCRL3* [64]	−110A/A −110A/G −110G/G	Associations with RA, SLE and autoimmune thyroiditis. Serum IgG4 levels positively correlated with number of susceptible alleles
*IKFZ1* [65]	c. 548G>A	Elevated IgE and IgG4, and allergic rhinitis. Combination with *UBRF4* variant thought to confer high penetrance to IgG4‐RD
*KCNA3* [66]	rs2840381G>A rs1058184A>C rs2640480C>A rs1319782C>T	Linked with AIP susceptibility
*MLL3* [60]	rs111493987C>A	Identified link with extra‐pancreatic lesions
*PAI1* (*SERPINE1*) [67] *PRSS1* [67] *PLG* [67]	c.‐816A>G c.292_293insC c.1465T>C	Ligneous conjunctivitis and recurrent pancreatitis
*P2RX3* [60]	c.195delG	Scleroderma, SLE, RA
*PRSS1* [68]	c.276G>T c.346C>T c.137C>T c.415T>A c.416G>T	Negatively associated with IgG4‐RD relapse. Hereditary pancreatitis caused by the PRSS1 gain of function mutation which gives rise to high (incomplete) disease penetrance [69]
*SPINK1* [68]	194 + 2(IVS3 + 2) TC	Increased susceptibility to chronic pancreatitis when combined with other factors [70]
*TBX21* [71]	c.466_471delGAGATGinsAGTTTA	Increased switching to IgG4
*TNF* promoter [62]	−863 C/A	Associated with extra‐pancreatic involvement
*TNIP1* [72]	p.Glu333Pro	First patient has Sjogren's disease‐like symptoms, the other patient presents with SLE
*TOP1* [60]	c.2007delG	Scleroderma, systemic lupus erythematosus (SLE), rheumatoid arthritis (RA)
*UBR4* [65]	c.12537T>A	Elevated IgE and IgG4, and allergic rhinitis. Combination with *UBRF4* variant thought to confer high penetrance to IgG4‐RD

### 1.8 Major Types of Immune Cells Involved in IgG4‐RD

#### 1.8.1 CD4 ^+^ Helper and Cytotoxic T Lymphocyte (CTL) Subsets

**FIGURE 2:**
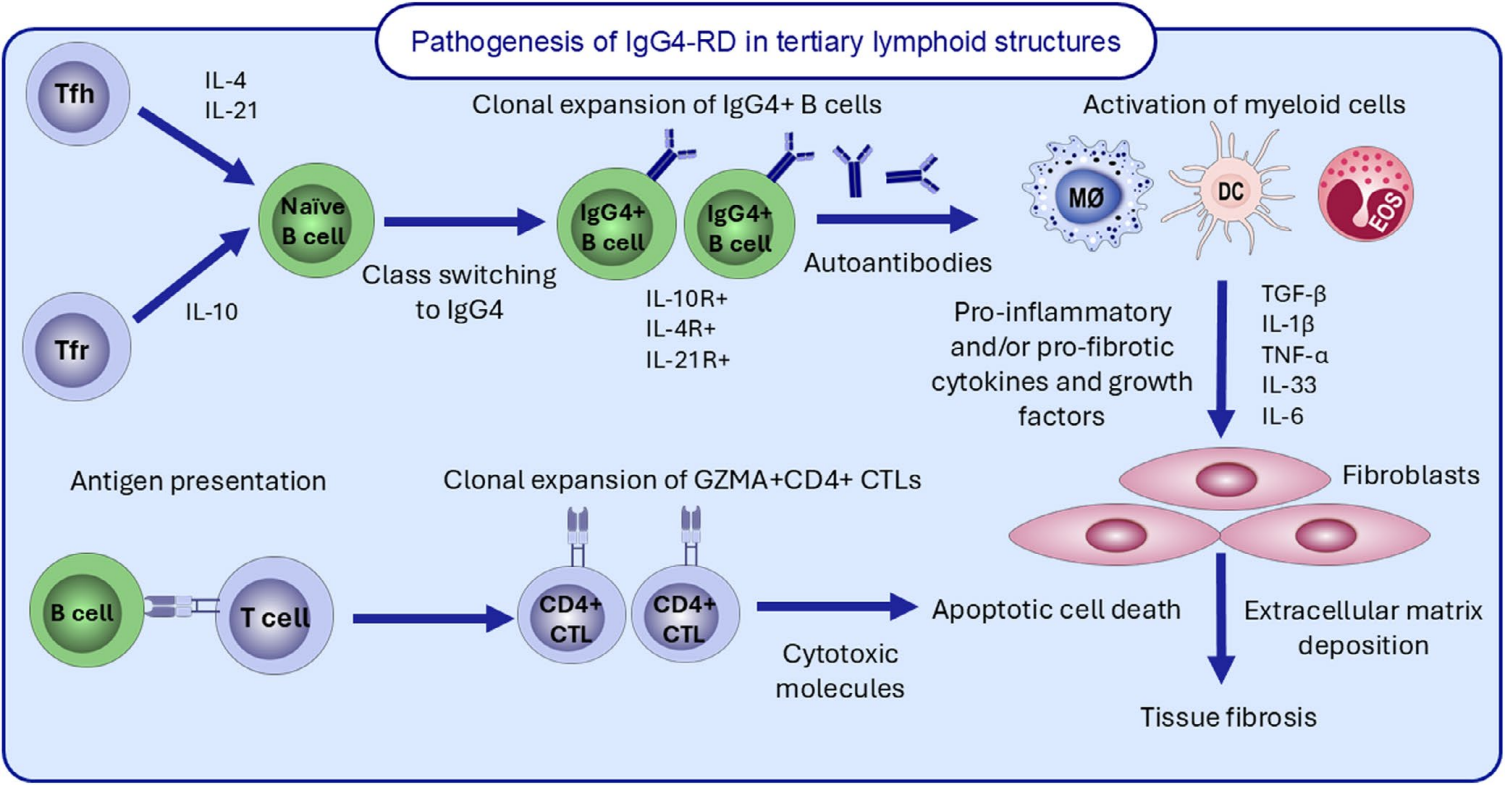
Proposed underlying pathogenic mechanisms of IgG4‐RD in tertiary lymphoid structures and other affected tissue sites involve IL‐4, IL‐21 and IL‐10, released by T follicular helper (Tfh) and T follicular regulatory (Tfr) cells. These cytokines drive the class‐switching of naïve B cells into IgG4‐expressing memory B cells and plasmablasts. These B cells then generate autoantibodies against a variety of autoantigens, potentially contributing to the activation of myeloid cells, including macrophages, eosinophils and dendritic cells. Antigen presentation by B cells also leads to the clonal expansion of cytotoxic CD4^+^ T cells. Collectively, autoantibodies, cytotoxic molecules, along with pro‐inflammatory and pro‐fibrotic soluble factors released by myeloid cells, drive tissue pathology, extracellular matrix deposition and fibrosis.

CD4^+^ T helper cells are one of the most abundant T‐cell subsets in IgG4‐RD affected tissues (Figure 2). Mattoo et al. first reported oligoclonal expansion of CD4^+^ CTLs in inflamed IgG4‐RD tissues [76]. CD4^+^ CTLs secrete pro‐fibrotic cytokines (IL‐1β, TGF‐β1, IFN‐γ) as well as cytolytic molecules (granzyme A, granzyme B, perforin) [77]. While CD4^+^ T cell populations were found to secrete higher levels of IFN‐γ in IgG4‐related AIP than in controls, IL‐4‐secreting T cells were not increased in these patients. This study also identified the CD4^+^ CTLs as SLAMF7^+^, a marker usually highly expressed on malignant cells [76]. SLAMF7^+^CD4^+^ CTLs have an unusual phenotype, equipped with cytolytic capabilities, secreting perforin and granzyme to kill target cells via MHCII molecules. B cell depletion via rituximab (RTX) produced a marked decrease in circulating CD4^+^SLAMF7^+^ CTLs, while having minimal to no effect on circulating naïve B cells, GATA3^+^, or FOXP3^+^ CD4^+^ T cells [76], suggesting CD4^+^ SLAMF7^+^ CTLs have a heavy dependency on B cells for their maintenance or survival. CD4^+^SLAMF7^+^ CTLs were abundant in both patients with and without atopy, as opposed to circulating GATA3^+^ Th2 memory cells being present in atopic patients alone [76]. Taken together, Th2 cells per se are unlikely to drive IgG4‐RD pathogenesis, but rather, are indicative of a long‐lived T‐cell memory response against a host of environmental allergens [76]. These preliminary findings emphasise that a clear Th2 skew has not been definitively established for the disease. Further research is needed to elucidate the precise roles of Th1 and Th2 responses in the pathogenesis of IgG4‐RD.

#### 1.8.2 CD8 ^+^ Memory T Cells

Frederich et al. proposed a novel role for CD8^+^ memory T cells as an effector arm of the IgG4‐related immune response [78]. The IgG4‐RD manifestation investigated was an intrathecal inflammatory pseudotumor (IPT) present in the space between the spinal cord and its protective membrane (theca). The strongest cell–cell interactions in the IPT were between pathogenic B cells and CD8^+^ effector memory T cells. The most highly expressed receptor for the B cell subset was CD74 and for the CD8^+^ T cells, its conjugate ligand macrophage inhibitory factor (MIF), which is recognised as a B‐cell chemokine and could encourage pathogenic B‐cell migration to affected tissues [78]. The CD74‐MIF interaction is implicated in other autoimmune diseases and cancer [79], and warrants further investigation in the context of IgG4‐RD. This study also found that IgG4‐plasma cell maturation is mediated by a cytotoxic subset of CD4^+^ T cells, which had an increased abundance in the brain parenchyma and cerebrospinal fluid compared with controls [78]. Crosstalk via the CCL5/CCR4/5 axis was identified between CD8^+^ memory T cells and CD4^+^ CTLs.

#### 1.8.3 T Follicular Helper (Tfh) Cells

Evidence increasingly suggests that Tfh cells drive IgG4 class switching and play a key role in IgG4‐RD pathogenesis. For instance, Kamekura et al. identified that Tfh cells account for 70% of the CD4^+^ T cells present within tertiary lymphoid structures, with Tfh2 cells being the predominant subtype [80]. Tfh2 cells within the lesion infiltrate express TIGIT, a marker indicating pronounced IL‐21 expression, promoting differentiation and class switching of IgG4^+^ B cells characteristic of IgG4‐RD [81]. Maehara et al. successfully purified cytokine‐secreting human Tfh cells from secondary and tertiary lymphoid structures [82]. Analyses of Tfh cell transcriptomes together with multicolour immunofluorescence from IgG4‐RD tissues showed that BATF^+^ (a transcription factor regulating IL‐4 secretion) Tfh cells had greater expansion in the IgG4‐RD cohort than in control secondary lymphoid organs [82]. Furthermore, these BATF^+^ IL‐4‐secreting Tfh cells in diseased tissue were highly correlated with patient serum IgG4 levels but not with the total serum level of IgA or IgE [82]. Therefore, it is proposed that this unique BATF^+^ Tfh cell subset may help to switch B cells to IgG4‐secreting short‐lived plasmablasts or plasma cells in patients with IgG4‐RD.

**FIGURE 3:**
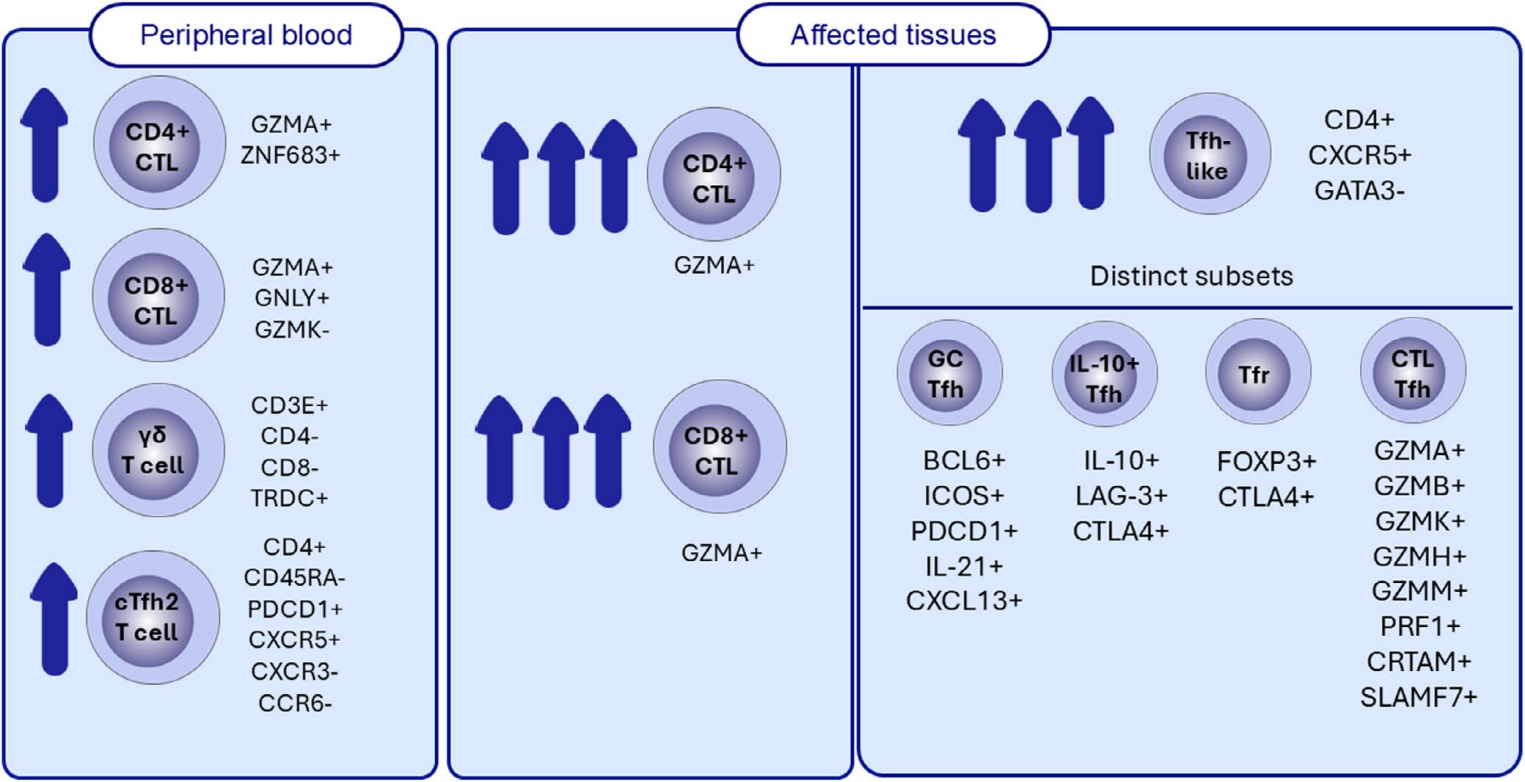
Detailed characterisation of sub‐types of T cells in peripheral blood [83] and tertiary lymphoid structures of IgG4‐RD based on scRNA‐seq or flow cytometric analysis [84, 85, 86]. GATA3‐CD4^+^CXCR5^+^ Tfh‐like cells were found to be located near class‐switching B cells. In addition, CD4^+^ GZMA^+^ CTLs were found to be highly expanded in tissue lesions.

Recently, single‐cell RNA sequencing (scRNA‐seq)‐based studies revealed notably expanded populations of different subtypes of cytotoxic T cells and Tfh cells in the peripheral blood or tertiary lymphoid organs of affected tissues from IgG4‐RD patients (Figure 3). Circulating Tfh (cTfh) cells are thought to be a key player in IgG4‐RD [87]. Three subsets of cTfh have been put forward: cTfh1, cTfh2 and cTfh17, which can secrete conventional T helper cell cytokines, namely IFNγ, IL‐4 and IL‐17. cTfh2 cells have been shown to induce IgG4‐class switching in B cells [88]. Notably, numbers of cTfh2 cells are increased in patients with active, untreated IgG4‐RD [89]. It has been suggested that this circulating subset reflects the phenotype of resident Tfh2 cells in the affected tissue environment. Studies have also associated PD‐1^hi^ cTfh2 cells with IgG4‐RD [90, 91]. scRNA‐seq has identified several subtypes of Tfh cells in tertiary lymphoid tissues of patients (Figure 3). This includes a previously unknown IL‐10^+^LAG3^+^ Tfh cells infiltrating IgG4‐RD affected submandibular glands [84]. Upregulation of canonical checkpoint inhibitors in these cells suggests post‐activation exhaustion and overactivated Tfh cell infiltrates may contribute to the systemic inflammation and fibrosis of IgG4‐RD [77]. All in all, further developments in scRNA‐seq and spatial transcriptomics will likely provide even greater detail about the causal roles and heterogeneity of these subsets in disease progression.

#### 1.8.4 Eosinophils and Group 2 Innate Lymphoid Cells (ILC2s)

There is an emerging body of evidence which suggests that eosinophils are associated with IgG4‐RD development and pathogenesis. Mild to moderate tissue eosinophilia is regarded to be a pathological hallmark of IgG4‐RD; 20%–40% of IgG4‐RD patients present with peripheral eosinophilia, which manifests as tissue eosinophilia in 51%–86% of these patients [92]. IgG4 serum levels and eosinophilia may be positively correlated as patients with elevated serum IgG4 levels also had higher blood absolute eosinophil counts [93]. Eosinophil pathology in IgG4‐RD may also be sex‐specific, with one prospective study showing male IgG4‐RD patients presenting with higher peripheral eosinophil counts, CRP and IgG4 serum levels at baseline, as opposed to their female counterparts [44]. Peripheral eosinophilia has also been reported as a risk factor for failure to induce remission in corticosteroid monotherapy [94], disease duration [95], number of involved organs [96], *de novo* organ involvement [97], as well as being an independent predictor for disease relapse [98]. Up to 40% of IgG4‐RD patients have been reported as having allergic disease [99]. However, Della Torre et al. argue that these atopy rates are similar in the US population at large. Interestingly, they found that most of their IgG4‐RD patient cohort were non‐atopic yet expressed high IgE and peripheral blood eosinophilia [100]. Puzzlingly, Saeki et al. described peripheral blood eosinophilia as not significantly different between both atopic and non‐atopic patients [101], and Zhang et al. concluded that eosinophilia appeared to be independent of allergy suggesting a multifactorial cause at play [95]. Unknown processes inherent to IgG4‐RD itself are thought to contribute to these uncharacteristic features [100]. Nonetheless, the consistent detection of both peripheral and tissue eosinophilia in IgG4‐RD holds significant prognostic value and underscores the need to investigate alternative therapeutic strategies further.

Eosinophils may also have interplay with other cell types, such as IL‐5‐producing ILC2s, which are considered a significant contributor to tissue eosinophilia in inflammatory disorders and can drive the process independently of functional T cells [102]. Interestingly, Zhang et al. found that the circulating ILC2 level in IgG4‐RD patients was significantly higher than that in healthy controls [103]. These findings suggest that ILC2s could be one of the inherent mechanisms of IgG4‐RD which contribute to eosinophilia observed in both atopic and non‐atopic patients. ILC2s can also activate macrophages via IL‐13 signalling, which promote their differentiation into profibrotic M2 macrophages. Ishiguro et al. found that IgG4‐RD patient‐derived M2 macrophages overexpressed the innate immunity receptor TLR7 [104]. Not only is TLR7 signalling associated with class switching of B cells in autoimmunity [105], but also with the production of profibrotic factors. The same group found IL‐33 expression to be positively correlated with TLR7 expression in salivary glands from IgG4‐RD patients.

#### 1.8.5 Other Innate Immune Cells in IgG4‐RD

The roles of various innate immune cells in IgG4‐RD are increasingly being studied. For instance, scRNA‐seq analysis of peripheral blood mononuclear cells (PBMCs) from IgG4‐RD patients revealed that different monocyte subsets significantly upregulate genes involved in immune response, chemotaxis and adhesion compared to healthy controls [83, 106]. Specifically, the expression levels of resistin (encoded by *RETN*, a pro‐inflammatory and pro‐fibrotic molecule) in classical monocytes (CD14^++^ FCGR3A^−^) and serum levels of resistin are significantly elevated in these patients [83]. Similarly, NK cells in the peripheral blood of IgG4‐RD patients also show upregulation of several genes associated with cell activation and cytotoxicity [83].

Plasmacytoid dendritic cells (pDCs) are known for their ability to produce large amounts of type I interferons. In patients with IgG4‐RD‐related autoimmune pancreatitis (AIP), IFN‐α and B‐cell activating factor (BAFF)‐expressing pDCs were identified in pancreatic tissue, with these patients also exhibiting higher serum IFN‐α and BAFF levels than healthy controls [107]. Additionally, pDCs from IgG4‐RD‐related AIP have been reported to produce IL‐33. All these soluble factors are thought to significantly contribute to disease progression [108]. Furthermore, in experimental mouse models of AIP/IgG4‐RD induced by polyinosinic‐polycytidylic acid (poly[I:C]) administration, interactions between T cells and pDCs, mediated by CXCL9, CXCL10 and CCL25, were reported to drive the disease. Notably, in human IgG4‐RD‐related AIP patients, serum levels of these soluble factors strongly correlate with disease extent [63]. Studies highlight the crucial role of pDCs in experimental AIP, showing that their activation is a key factor. This activation can be triggered by innate immune responses to intestinal microflora and exacerbated by gut dysbiosis and intestinal barrier dysfunction, the latter leading to bacterial translocation (e.g., *Staphylococcus sciuri* ) into the pancreas, thus worsening AIP [109, 110, 111]. Finally, in the peripheral blood of IgG4‐RD patients, both conventional DCs and pDCs were observed to upregulate genes involved in chemotaxis, inflammation and immune response [83].

The roles of mast cells in IgG4‐RD are not yet extensively studied. One study reported increased levels of IL‐13‐producing mast cells (based on c‐kit staining) in submandibular gland tissue samples from IgG4‐RD patients compared to normal glands [112]. Another study observed elevated mast cells expressing cytoplasmic IgE and high‐affinity IgE receptor FcεRI in tissue samples from IgG4‐related lymphadenopathy compared to non‐specific lymphoid hyperplasia, suggesting the possible internalisation of the IgE and FcεRI when compared to non‐specific lymphoid hyperplasia [113]. While scRNA‐seq analysis of submandibular glands from IgG4‐RD patients and controls revealed gene expression differences in all immune and stromal cell types, including mast cells [114], no significant difference in mast cell frequency was observed between patients and controls. In the same study, macrophages were identified as an abundant cell type, showing upregulation of genes such as *SPP1* (coding for secreted osteopontin) and many other genes involved in phagosome function and antigen presentation [114].

Mer receptor tyrosine kinase (MerTK), a negative regulator of the immune system, plays a crucial role in wound healing, phagocytosis of apoptotic cells and fibrosis. MerTK^+^ macrophages have been proposed as a novel contributor to the pathophysiology of IgG4‐RD [115]. These macrophages not only significantly infiltrate IgG4‐RD lesions, but they may also interact with ProS1‐expressing lymphocytes to sustain fibrosis within these lesions. ProS1 is the primary MerTK ligand found on apoptotic cells and activated lymphocytes in IgG4‐RD lesions. A physical interaction between ProS1‐expressing cells and MerTK^+^ lymphocytes has been observed in affected tissues, and these interactions are likely to drive TGF‐β production. Interestingly, the number of these MerTK^+^ macrophages decreases following B cell depletion therapy. This reduction highlights MerTK^+^ macrophages as a relevant cell population in the pathogenic microenvironment of IgG4‐RD [115].

### 1.9 Fibrosis and Cytokines

Emerging evidence points to pro‐fibrotic pathways contributing to disease pathogenesis in IgG4‐RD. The IL33/ST2 axis may play a crucial role in promoting tissue fibrosis in this disease. IL33 is highly expressed in epithelial cells and can be induced in several immune cells, acting as an alarmin to signal tissue damage [116]. This axis can drive the expansion of Tregs, promote M2 macrophage polarisation and stimulate IL13 secretion from ILC2s [117]. IL‐33 further amplifies the fibrotic effect by upregulating IL13 in Th2 cells, mast cells, basophils and eosinophils [118]. Moreover, a recent IgG4‐RD cohort study found that hyperactive ILC2s secrete increased IL9 via IL33/ST2 stimulation, potentially promoting fibrosis through Treg activation and profibrotic TGFβ induction [103]. Although most IL33/ST2 studies rely on isolated organ systems (e.g., pulmonary fibrosis models), further clinical research is needed to clarify whether this axis is the core driver of Th2‐mediated fibrosis in IgG4‐RD.

The IL6/IL6R axis also significantly contributes to fibrosis in IgG4‐RD by inducing stromal fibroblasts to adopt a lymphoid tissue organiser (LTo) phenotype. LTo's express IL7, which supports tertiary lymphoid structure formation and immune cell recruitment [116]. Elevated levels of IL6 and IL6R in patient serum and tissues underscore the proinflammatory properties of fibroblasts; indeed, IL6/IL6R transsignalling in these cells enhances the release of cytokines such as IL7, IL12, IL23 and BAFF [119]. Increased fibroblast‐derived cytokine production can modulate immunoinflammatory processes and contribute to tissue fibrosis. BAFF and the proliferation‐inducing ligand APRIL are essential for circulating B cell maturation, survival and maintenance [120], with their elevated levels linked to B cell pathologies observed in conditions like SLE, Sjögren's syndrome and RA [121]. The role of fibroblast signalling in tertiary lymphoid structure formation thus merits further scrutiny in IgG4‐RD pathogenesis.

### 1.10 Mouse Models

IgG4 regulation studies in vivo have been disadvantaged due to the absence of IgG4 in mice [122]. Two approaches have been used to tackle this issue. Cevhertas et al. proposed that a humanised NOD‐*scid Il2rɣ* ^*null*^ (NSG) mouse model can be used to study IgG4 regulation using engraftment of human CD45^+^ cells [122]. Intraperitoneal injection of IL‐10 at Days 3 and 12 post‐engraftment produced a significant increase in the serum levels of IgG4 in comparison with all other human immunoglobulin isotypes. Gon et al., on the other hand, have generated a human *IGHG4* knock‐in model using MRL/lpr mice [123]. Not only did these mice show high serum levels of the human IgG4 and increased populations of IgG4^+^ plasma cells and T cell infiltrates in the spleen, but they also aggravated systemic organ inflammation. This included diffuse inflammatory cell infiltration in the salivary gland, a common manifestation in IgG4‐RD [124]. Joachim et al. also put forward a mouse model which can be used to study the aetiology of IgG4‐RD [125]. *Lat* ^*Y136F*^ mice have a loss of function mutation in the linker for activation of T cells (LAT) adaptor and develop defective LAT signalosome pathology (DLSP), an autoimmune disorder with type 2 inflammation. A unique feature of this disease is that the persistent inflammation does not require constant T cell input. Instead, upon first engagement with self‐antigen, the autoreactive T cell receptors relinquish their role in T cell activation completely to the CD28 costimulatory molecules, leading to widespread B cell activation. *Lat* ^*Y136F*^ mice spleen and lung scRNASeq produced a cell constellation entailing all cell types regarded as causative of IgG4‐RD. These include Tfh cells, CD4^+^ CTLs, activated B cells and plasma cells. Sun et al. used the *Lat* ^*Y136F*^ mice as their in vivo IgG4‐RD model to explore an alternative therapeutic to monoclonal antibody B‐cell blockade. This group engineered anti‐CD19 CAR‐T cells from IgG4‐RD patients and injected them into *Lat* ^*Y136F*^ mice. The mice showed sustained B‐cell depletion as well as reduced inflammatory lesions and fibrosis [126]. *Lat* ^*Y136F*^ mice are a promising disease model displaying several characteristic features of IgG4‐RD with an expanded capacity to provide robust validation of emerging therapeutics in vivo.

### 1.11 IgG4‐RD Mimickers

**FIGURE 4:**
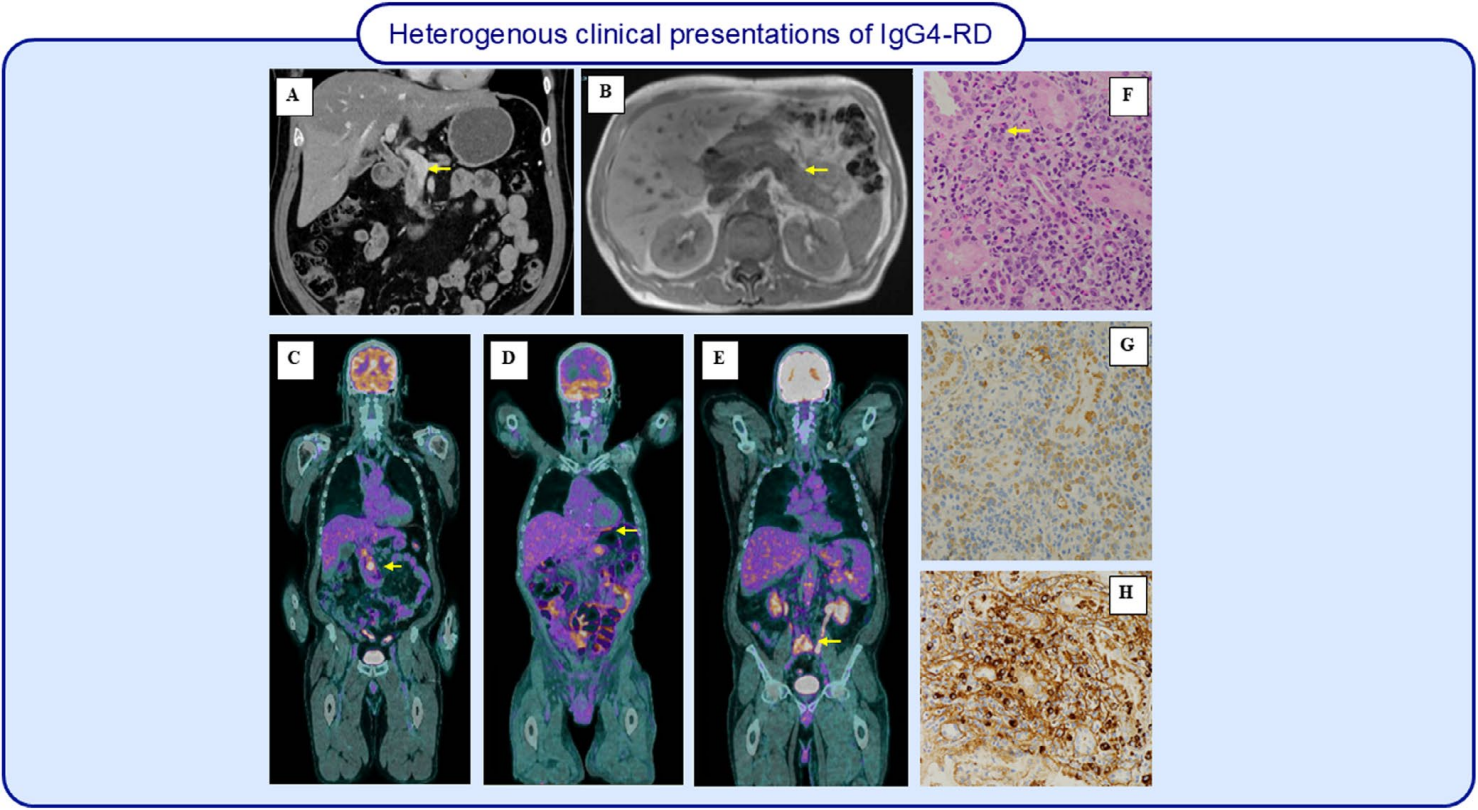
Imaging and histopathology of IgG4 disease demonstrating heterogeneous clinical presentations (A–E) and histopathology (F–H). (A–C) FDG PET Scan, (A) Demonstrates FDG‐avidity in the pancreatic head/uncinate process suggestive of pancreatic IgG4‐RD. (B) FDG‐avidity in the pericardial region in a patient with known IgG4 pericardial disease. (C) Increased activity seen in association with abnormal soft tissue stranding in the retroperitoneum, around the distal inferior vena cava (IVC) and aorta, bilateral common iliac and adjacent to the left internal iliac vessels, in keeping with active retroperitoneal fibrosis. (D) Computed Tomography (CT) scan of the abdomen and pelvis demonstrating extrahepatic bile duct thickening with mild intrahepatic biliary dilation in the right lower lobe suggestive of IgG4‐related cholangitis. (E) MRI of the abdomen demonstrating diffusely T2‐hypointense pancreatic parenchyma with sausage‐shaped morphology suggestive of IgG4‐RD.

IgG4‐RD can mimic other diseases with elevated IgG4 levels, peripheral eosinophilia and tumour‐like masses. One such mimic is primary sclerosing cholangitis, which presents with bile duct thickening (Figure 4A) and elevated IgG4 [127]. Systemic inflammatory conditions with elevated IgG4 levels, such as retroperitoneal fibrosis (Figure 4B) and multicentric Castleman disease [128], are also effective mimickers of IgG4‐RD. Lymphadenopathy due to chronic inflammation is also another diagnostic mimic. Hard fibrotic thyroid glands presenting as thyroid carcinoma mimic IgG4‐related thyroid disease. Salivary gland enlargement can be observed in Sjogren's syndrome and/or swollen lachrymal glands seen in Mikulicz's disease that are commonly confused with IgG4‐related dacryoadenitis and sialadenitis. IgG4‐RD mass‐like presentations appear brightly on PET scans (Figure 4C–E) due to high uptake of ^18^F‐FDG, which is generally indicative of cancer [129]. Plasma cell neoplasms (which can be either malignant or benign) are one such condition that mimics IgG4‐RD. Both conditions are characterised by elevated plasma cells and show increased uptake on PET scans. Hyper‐eosinophilic syndromes, including EGPA [130], can mimic IgG4‐RD. While mimickers remain a challenge in the diagnosis of IgG4‐RD, improved diagnostic criteria and guidelines aim to reduce the incidence of incorrect diagnoses and improper medical interventions.

### 1.12 Biomarkers

Establishing a biomarker in IgG4‐RD is difficult due to the disease heterogeneity including organ pathology, symptomatology and ancillary laboratory parameters. Despite this, several biomarkers have been put forward, with varying specificity, sensitivity and abundancy across manifestations. Serum IgG4 levels are widely used as a biomarker for IgG4‐RD, and 70% of patients have increased serum levels [14], which can support the diagnosis in the appropriate clinical context. Elevated IgG4 levels, especially when combined with elevated IgE, can help differentiate IgG4‐RD from other diseases, such as Sjögren's syndrome [41]. However, levels of IgG4 cannot serve as a lone diagnostic marker. Indeed, four out of five patients with elevated IgG4 serum levels enrolled in a prospective longitudinal study from the UK did not meet IgG4‐RD diagnostic criteria [131]. Notably, elevated IgG4 levels can also be detected in various other AID, as well as a broad spectrum of infections, malignancies, pancreato‐biliary, hepatic and respiratory diseases [132].

Multi‐ethnic cohorts also present challenges, as Asians have been shown to have higher serum IgG4 levels compared to non‐Asians [133]. However, there are some prominent candidates for novel biomarkers. Increased levels of CD19^low^CD20^−^CD27^+^CD38^+^ cells (circulating plasmablasts) are reported in IgG4‐RD patients with active disease whose serum IgG4 concentrations are normal [134]. A proteomics approach detected galectin‐3 overexpression (a 13‐fold change from controls) in the stroma and myofibroblasts of IgG4‐related pancreatitis samples [135]. Autoantibodies against galectin‐3 were also found to be elevated in 45 US IgG4‐RD patients [136] and laminin 5–11 E8 IgG autoantibodies were identified in 51% of a small Japanese IgG4‐RD cohort [137]. An emerging body of evidence supports the pro‐fibrotic cytokine CCL18 as a useful biomarker for evaluating both IgG4‐RD disease activity and response to therapy. Specific upregulation of CCL18 in B cells, CD11c^+^ (age‐associated) T cells and macrophages has been identified within IgG4‐RD salivary gland manifestations [138].

### 1.13 Therapeutics

Corticosteroids remain the first‐line treatment for IgG4‐RD, with the goal of inducing remission. The initial recommended dose of 0.6 mg/kg/day oral prednisolone for 2–4 weeks, followed by maintenance with low doses over a 3‐ to 6‐month period, is recommended to prevent relapse [139]. Maintenance therapy with low‐dose prednisolone over a period of 2–3 months is recommended to prevent relapse [140]. However, the specific dosing and tapering regimen vary across different guidelines [141]. While patients respond well to corticosteroids initially, relapse rates are relatively high. Previous studies have indicated relapse ranges from 23% to 58% depending on disease characteristics and treatment regimen [142]. Furthermore, prolonged courses can lead to steroid‐related toxicities. Hence, modern treatment regimens prefer combination or steroid‐sparing therapies over glucocorticoids alone. Although csDMARDs, such as mycophenolate mofetil, have been used as steroid‐sparing therapies, their evidence in IgG4‐RD is weak, as efficacy is primarily supported by case reports [143].

B cells (particularly long‐lived plasmablasts) are considered key players in disease pathogenesis, providing a robust set of clinically relevant markers as a platform for novel B cell depletion therapies. RTX, an anti‐CD20 monoclonal antibody, has been reported to be an effective steroid‐sparing agent for patient remission. Successful remission induction with RTX showed marked reduction in plasmablast counts [144], a disease relapse predictor. While RTX has a favourable safety profile over csDMARDs and other immunomodulators [145], long‐term CD20 B‐cell depletion is associated with an increased incidence of infection, which may carry over to CD19‐targeted B cell depletion. The recent success of inebilizumab, a recently FDA‐approved anti‐CD19 monoclonal with an 87% disease flare reduction in a Phase III trial [18], marks a significant advancement in managing IgG4‐RD, offering effective alternatives to corticosteroids and hope for reduced side effects and improved disease control. The persistence of long‐lived plasma cells and T cell‐driven pathogenesis poses challenges for the B cell depletion approach, as some patients remain unresponsive to these therapies. Small molecule inhibitors provide therapeutic hope for B cell depletion‐resistant patients by targeting alternative pathways. These include inhibitors of BTK (rilzabrutinib, zanubrutinib), SLAMF7 (elotuzumab), JAK (baricitinib, tofacitinib), among other small molecule inhibitors. Immunomodulators have also been put forward, particularly for the management of disease relapse. In a recent multicentre trial, thalidomide was shown to effectively prevent relapse in IgG4‐RD, with the authors contending that the therapeutic advantages of thalidomide outweigh its side effects [146]. Thalidomide also showed promise as a steroid‐sparing agent, as well as markedly reducing serum IgG4 levels compared with the steroid‐plus‐placebo control group. Combination therapies with both immunomodulators and targeted therapies also provide a promising approach for refractory disease. Daratumumab (a CD38 targeted therapy), in combination with lenalidomide and dexamethasone, provided a successful treatment regimen for a systemic relapse case that was unresponsive to both corticosteroid and RTX therapy [147]. Table 3 summarises the current and emerging therapeutics for IgG4‐RD, including their intended target and desired effect, impact on serum IgG4 levels (if known) and their consideration as a steroid‐sparing therapeutic.

**TABLE 3:** Current and emerging therapeutics for the management of IgG4‐RD.

Drug	Target	Serum IgG4	Steroid‐sparing	Trial stage
Abatacept	CTLA‐4	Partial [148]	Probable (RA) [149]	Completed [148] Phase II NCT03669861
Baricitinib	JAK	Reduced [150]	Y (RA) [151]	Active, recruiting [152] NCT05781516
Belimumab	BAFF	Unspecified	Y (SLE) [153]	Results pending [121] Phase IV NCT04660565
CM310	IL‐4Ra	Unspecified	Unspecified	Not yet recruiting NCT05728684
Daratumumab	CD38	Reduced [147] (gamma‐globulins)	Effective as combination therapy [147]	None registered
Dupilumab	IL‐4Rα	Reduced	Y	Case report [154]
Elotuzumab	SLAMF7	Unspecified	Unspecified	Terminated [155] Phase II NCT0491847
Inebilizumab	CD19	Y [18]	Reduced [18]	Completed [18] Phase III NCT04540497
Mycophenolate mofetil	IMPDH	Unspecified	Effective as combination therapy [156]	Unknown [156], Phase II NCT05974683
Obexelimab	CD19	Unspecified	Unspecified	Active [157], recruiting Phase III NCT05662241
PRG‐1801	BCMA	Unspecified	Unspecified	Active [158], recruiting Early Phase 1 NCT06497387
PRG23‐11	BCMA + CD19	Unspecified	Unspecified	Active [159], recruiting Early Phase 1 NCT06497361 Active [159], recruiting Phase II NCT06794008
Rilzabrutinib	BTK	Unspecified	Y (pemphigus vulgaris and thrombocytopenia [160])	Completed [161] Phase IIa NCT04520451
Rituximab	CD20	Reduced [32]	Y [35]	Multiple trials [162]
Sirolimus	mTOR	Unspecified	Reduced (dermatomyositis [163]/retroperitoneal fibrosis [164])	Active, non‐recruiting [159] NCT05746689
Thalidomide	CRL4‐CRBN downstream signalling	Y [146]	Y [146]	None registered
Tocilizumab	IL‐6R	Reduced	Y	Case report [165], prospective cohort study [166]
Tofacitinib	JAK	Reduced [165]	Y [165]	Active, recruiting [167] NCT05625581
Zanubrutinib	BTK	Unspecified	Unspecified	Active, non‐recruiting [168] Phase II NCT04602598

### 1.14 Future Directions

Despite the progress made with IgG4‐RD diagnosis, treatment and awareness, major outstanding research questions and challenges remain in understanding and managing the disease. Data on comorbidities and modifiable risk factors for IgG4‐RD are sparse. Phenotypic differences in disease severity and prevalent manifestations across global cohorts warrant additional consideration to identify potential genetic or environmental factors contributing to these differences. Moving forward, it is crucial to leverage the power of multi‐omics technologies to further elucidate the roles of both immune and non‐immune cell subsets in disease pathogenesis. A deeper exploration of pro‐fibrotic pathways and associated cytokines will be essential to reveal the extent of their impact on disease progression. While significant progress has been made in managing IgG4‐RD over the past decades, particularly in addressing relapse, targeted therapies such as B cell depletion and immunomodulation offer promising therapeutic approaches to mitigate steroid‐related toxicity. There is a continued need to establish safer and shorter treatment regimens, as well as innovative strategies to capture patient cohorts unresponsive to B cell depletion. Future treatments must address the complexities of IgG4‐RD management to provide safer, more personalised treatment options and ultimately improve the lives of individuals with this condition.

## 2 Conclusion

While significant strides have been made in the diagnosis, treatment and general understanding of IgG4‐RD, this review underscores the profound molecular complexity and remaining challenges in disease management. Despite progress in identifying key immune cell populations and the development of targeted therapies, the precise aetiology, heterogeneous clinical presentations and detailed pathogenic mechanisms of IgG4‐RD require further in‐depth investigation. Ultimately, gaining a deeper understanding of IgG4‐RD pathophysiology will be essential to developing more effective and personalised therapeutic strategies, thereby significantly improving patient outcomes and reducing the substantial burden of this increasingly recognised fibroinflammatory disorder.

## Author Contributions

All authors contributed to the writing of this review manuscript, provided critical feedback and took responsibility for the accuracy of the data cited. All authors took final responsibility to submit for publication.

## Conflicts of Interest

The authors declare no conflicts of interest.

## Data Availability

The authors have nothing to report.
